# The Immunologic Response to *Trichophyton Rubrum* in Lower Extremity Fungal Infections

**DOI:** 10.3390/jof1020130

**Published:** 2015-07-17

**Authors:** Matthew S. Blutfield, Jenna M. Lohre, Derek A. Pawich, Tracey C. Vlahovic

**Affiliations:** Temple University School of Podiatric Medicine, 148 N. 8th St., Philadelphia, PA 19107, USA; E-Mails: tue53809@temple.edu (M.S.B.); tuf77759@temple.edu (J.M.L.); tue81439@temple.edu (D.A.P.)

**Keywords:** *Trichophyton rubrum*, dermatophytosis, cell-mediated immune response, toll-like receptor, keratinocyte, macrophage, dendritic cell, fungal infection, tinea pedis

## Abstract

Manifestations of *Trichophyton rubrum* infestations, such as tinea pedis, tinea cruris, and tinea corporis, are among the most common human skin diseases seen throughout the world. About 80% of patients presenting with acute dermatophytosis respond well to topical antifungal treatment. However, the remaining 20% of patients progress into a chronic state of dermatophytosis, which is resistant to antifungal treatment. Therefore, it is necessary to have a better understanding and appreciation for the diverse immune responses to Trichophyton as this is critical for the development of therapeutic strategies for those individuals who suffer from a chronic manifestation of *Trichophyton rubrum* (*T. rubrum*) infection. As a result, a comprehensive literature review was conducted to review and discuss previous studies that evaluated the human body’s defense to *T. rubrum* infections and to understand why and how these fungal infections invade the host defense system. Our research revealed that a cell-mediated immune response is critical in defending the body against *T. rubrum*. However, this organism has mechanisms that enable it to evade the immune system. Therefore, a more successful treatment for chronic *T. rubrum* infection would involve targeting the mechanisms of *T. rubrum* that diminish the immune response, while restoring the cell-mediated immune response.

## 1. Introduction

*Trichophyton rubrum* (*T. rubrum*) is a dermatophyte responsible for causing the majority of superficial fungal infections worldwide [1]. Dermatophytes are a subset of fungi that have the ability to invade keratinized tissues, such as skin, hair, and nails. This group of fungi can cause infection anywhere on the skin, however, they most commonly affect the feet, inguinal region, axillae, scalp, and nails [2]. The infection results in mild to moderate dermatological symptoms, with a range of severity of infection. Such variations are believed to be a result of the host’s immune response to the microorganism. This response is elicited by the keratinocytes, which are the first line of defense against microorganisms, such as *T. rubrum*. Several Toll-like receptors, such as TLR2, TLR4, TLR6, and Human Beta Defensin (HBD)-1, HBD-2, IL-1B, and IL-8, are expressed as part of the initial host defense [1]. Manifestations of *T. rubrum* infestations, such as tinea pedis, tinea cruris, and tinea corporis, are among the most common human skin diseases seen throughout the world. About 80% of patients presenting with acute dermatophytosis respond well to topical antifungal treatment. However, the remaining 20% progress into a chronic state of dermatophytosis, which is resistant to antifungal treatment [3].

A unique feature of the immune response to *T. rubrum* is that it has the capacity to elicit either an immediate (IH) or delayed type hypersensitivity (DTH) response. This is dependent on the host, and the host’s previous exposure to the antigen. Generally, the DTH response is associated with acute infections, with increased amounts of inflammation. The IH response is associated with chronic dermatophytosis infections. Symptomatically, induration is the hallmark of a DTH response. A local wheal and flare reaction is the hallmark of the IH response. It is unusual for an antigen, such as *T. rubrum* to cause either reaction, and this dichotomy is not fully understood. This irregularity in immune response has been suggested to be caused by the different enzyme profiles that the varying Trichophyton species produce, including lipases, alkaline phosphatases, esterases, *etc*. More importantly, this dichotomy helps to define the human immune system [4].

The diverse clinical responses to infection are now suggested to be related to different T-cell responses and differences in Helper T (Th) cell responses to common antigens. This draws the distinction between those suffering from a chronic *versus* acute dermatophytic infections [4]. *T. rubrum* is unique, in that it has an adaptive mechanism that helps it to avoid antagonizing the immune system of its human host. Therefore, unlike its zoophilic cousin *Trichophyton mentagrophytes*, *T. rubrum* is less likely to induce an intense delayed type hypersensitivity reaction, which was previously stated to be the trademark of an acute infection. As a result, it can be argued that patients who present with a chronic *T. rubrum* infection, possibly contain a defective cellular immune response. Moreover, these observations suggest that activation of T lymphocytes is critical in recovering from a dermatophyte infection. In a normal immune system, major histocompatibility complex (MHC) class I molecules present extracellular antigens to the CD8+ T cells, initiating a cytotoxic response [2].

The key to the host’s response to *Trichophyton rubrum* is its ability to recognize the pathogen as a foreign invader. Host-parasite interactions in dermatophyte infections are a balance between the pathogenicity of the fungi and the defenses of the host. Recognition of the parasite is the first step in the immune response to the pathogen. The Pattern recognizing receptors (PRRs), which recognize pathogen associated molecular patterns (PAMPS) are critical in achieving this. Interaction between the PRRs and PAMPs results in a release of cytokines, which induces an immune response. PAMPs include the mannans located in the yeast cell and many bacterial cell wall components, such as formylated peptides. Macrophages and dendritic cells express a mannose receptor (MR; CD206) that recognizes some organisms, including fungi-like *Candida albicans*, *Cryptococcus neoformans*, and *Pneumocystis jiroveci*. MR is able to recognize mannose, *N*-acetylglucosamine, and glucose in a calcium-dependent manner. Upon recognition of fungus and its associated carbohydrates, MR induces NF-κB activation and production of certain cytokines, including IL-1β, IL-6, IL-12, and GM-CSF. In addition, the MR induces phagocytes to phagocytose the pathogen. Nevertheless, some species of fungi, including certain strains of *Candida* and *Cryptococcus*, are able to evade the MR despite containing mannan within their cell walls. Therefore, they are able to induce an infection with little resistance from an immune response [2]. Surface glycoconjugates are also pivotal in mounting an immune response. These carbohydrate binding adhesins on the surface of the fungi interact with epithelial cells and macrophages, and may be key to the organism’s ability to invade its host. The interaction of carbohydrate binding proteins is likely responsible for recognition of *T. rubrum* in its mammalian host. Lectins on the pathogen likely interact with sugar on host epithelial cells, creating the fungi-host interface [5].

The purpose of this literature review is to evaluate and discuss previous studies that examine the body’s defense to *T. rubrum* infections and to understand why and how these fungal infections invade the host defense system. Obtaining a comprehensive understanding of the immune response to *T. rubrum* is critical to developing new drugs and treatment regimens for patients affected by dermatophytosis.

## 2. Experimental Section

Garcia-Madrid, Huizar-López, Flores-Romo, and Islas-Rodríguez [1] conducted a study that consisted of taking primary keratinocyte cultures from surgical specimens of human abdominal skin and exposing them to whole conidia or conidial homogenates. The whole conidia and conidial homogenates were prepared from a *T. rubrum* strain that was isolated from a patient with a tinea pedis infection, commonly known as “Athlete’s foot”. This dermatophyte sample was cultured in dextrose sabouraud agar for 15 days at 25 °C. Microconidia were collected from the agar and conidial homogenates were prepared by sonication. The keratinocytes were cultured in six-well plates and were either stimulated or unstimulated with 100 μg/mL lipopolysaccharides (LPS) from *E.coli*, 100 μg/mL lipoteichoic acid (LTA) from *S. aureus*, *T. rubrum* microconidia at a fungal cell/human keratinocyte ratio of 10:1, or with conidial homogenate of the same amount of conidia for 6, 18, 24, or 48 h. After 6 h of stimulation, cells were stained with Alexa labeled 488-labelled anti-TLR2, Alexa 647-labelled anti-TLR4, or FITC-labelled anti-TLR6 and their corresponding isolated controls. These cells were then collected via flow cytometry. The keratinocytes underwent immunofluorescence staining with dilutions of primary antibodies of polyclonal goat anti human HBD-1, polyclonal goat anti human HBD-2 or goat normal serum, which served as a negative control. Concentrations of IL-1B and IL-8 in the cell culture supernatants were determined by ELISA. The results of the study showed that keratinocytes do indeed recognize and respond to cell wall components of *T. rubrum*. Furthermore, after being stimulated by the microbe, the conidia induced an increase in size, number, and cytoplasmic complexity of the keratinocytes. The whole conidia affected the immune response by inhibiting the TLR expression and HBD synthesis. The homogenate conidia had the opposite effect by actually increasing the expression of the TLRs and HBD-1 and HBD-2. Neither the whole conidia nor the homogenate conidia had any effect on the IL-1B and IL-8 [1].

A study by Waldman, Segal, Berdicevsky, and Gilhar [3] was conducted to determine if a similar T cell antimicrobial was noted in the presence of dermatophyte pathogens. Their study aimed to investigate the fungicidal mechanism of the T cells in chronic dermatophyte infections *versus* a normal population. Fifteen patients with chronic dermatophytosis and fifteen normal healthy patients with a history of acute dermatophytosis were enrolled. The chronic patient subset included eight males and seven females between the ages of 20 and 60 years old. Of these patients, five had tinea corporis, seven had tinea pedis, and three had tinea manuum. Thirteen of these patients were infected by *T. rubrum* and the other two patients were infected by *T. mentagrophytes*. The healthy volunteers included eight males and seven females also between the ages of 20 and 60 years old. Seven of these patients had a history of tinea pedis, six had a history of tinea manuum, and two had a history of tinea corporis. *T. rubrum* was the cause of infection in ten of these patients, while *T. mentagrophytes* caused infection in the other five patients. Dermatophytes were isolated from the chronically infected patients and cultured onto sabouraud media. Human peripheral blood mononuclear cells (PBMC) were isolated from both the chronic dermatophytic patients and the healthy patients. T lymphocytes were cultured and then restimulated with a nonspecific mitogen, such as phytohemagglutinin (PHA), trichophyton, dermatophytes homogenate or IL-2 alone. During the study, the researchers observed a difference in the proliferative rate of T lymphocytes between patients with chronic dermatophytosis and healthy patients. There was a higher proliferation of T lymphocytes noted in the healthy patients as opposed to the chronic patients. The highest T lymphocyte proliferation rate was observed in the *T. mentagrophyte* patients, followed by the *T. rubrum* patients. There was also an observed decrease in T lymphocyte proliferation following stimulation by the dermatophyte homogenate. This decrease in T lymphocyte proliferation was not observed when stimulated by a nonspecific mitogen. The study also showed higher cytotoxic activity of T lymphocytes against both *T. mentagrophytes* and *T. rubrum* in healthy patients compared to the chronic patients. Moreover, T lymphocytes demonstrated a greater cytotoxic response to *T. mentagrophytes* compared to *T. rubrum* in both patient populations. Nevertheless, the study showed that the chronic patients had a significantly depressed cytotoxic response of T lymphocyte toward dermatophytes. In the study, the researchers also observed the type of damage induced by the T lymphocytes toward the dermatophytes. In healthy patients, cultures revealed massive destruction of hyphae, striking lyses and breaking of hyphae along attachment points with the lymphocytes. These signs of destruction were seen against both *T. mentagrophytes* and *T. rubrum*. Significantly less lysis was observed in chronic patients [3].

Bressani, Santi, Domingues-Ferreira, Almeida, Duarte, and Moraes-Vasconcelos [2] evaluated the lymphoproliferative capability and the synthesis of cytokines from twenty patients presenting with extensive dermatophytoses. The evaluation of T, B, and NK cells phenotype of patients’ lymphocytes was performed through flow cytometry. In addition, the researchers observed the lymphoproliferative response to phytohaemagglutinin (PHA), anti-CD3 (OKT3), pokeweed mitogen (PWM), Candida species (CMA), extract of *T. rubrum*, and the main fungal epitope TriR2 (T). These immune responses were observed via polyacrylamide gel electrophoresis (SDS-PAGE). They also evaluated IL-4, IL-10, IL-12, and IFN-γ using a capture immunoassay method (ELISA). Among the patients with extensive dermatophytosis, only two obtained positive responses to the *T. rubrum* extract. The same amount of T, B, C, and NK cells were observed between the patients and controls. Higher concentrations of carbohydrates in some extracts may have reflected the presence of mannans, which have the ability to suppress the cellular response in vitro. The lymphoproliferation test showed significant differences between groups stimulated by PWM, CMA, and TriR2 [2].

Esquenazi, Alviano, De Souza, and Rozental [5] conducted a study to gain an appreciation for the role of surface glycoconjugates in cell adhesion during *T. rubrum* infections. These carbohydrate binding adhesins on the fungi’s surface interact with epithelial cells and macrophages, and may be key to the organism’s ability to invade its host. The researchers hypothesized that the interaction of carbohydrate binding proteins are likely responsible for recognition of *T. rubrum* in its mammalian host. Lectins on the pathogen likely interact with sugar on host epithelial cells, creating the fungi-host interface. The author’s study involved observing Chinese hamster ovary epithelial cells. These epithelial cells express sialic acid on their surface. Mutant strains were selected based on resistance to cytotoxic plant lectins and each strain expressed different surface carbohydrates. Lec1 mutants expressed mannose, Lec2 mutants expressed galactose, and Lec8 mutants expressed *N*-acetylglucosamine. Macrophages were treated with cytochalasin D, in order to block their phagocytic activity and determine whether *T. rubrum* could invade non-phagocytic cells. Neo Glycoproteins were labeled with fluorescein and colloidal gold in order to confirm the existence of lectin like adhesins on the surface of microconidia of *T. rubrum*. Results were measured using electron microscopy and flow cytometry. The results showed a greater adhesion to Lec1 and Lec2 mutants. *T. rubrum* attached to and was ingested by all cell lines tested. Cells preincubated with sodium periodate decreased both adhesion and endocytic index, proving the importance of carbohydrates in the interaction process. It was confirmed that the fungi could infect non-phagocytic cells. The tinea penetrated into hamster cells using macrophages treated with cytochalasin D. Cytometric analysis confirmed that the fungi recognized mannose and galactose. *T. rubrum* binding was inhibited by the addition of methyl α-d-mannopyranoside and methyl α-d-galactopyranoside. Heating of the cells also reduced binding. This further suggests a glycoprotein structure of the adhesins. Electron microscopy confirmed lectin-like molecules in the fungal cells [5].

Santiago, Bomfim, Criado, and Almeida [6] conducted multiple studies that evaluated the immune response to *T. rubrum* conidia. Their first study observed the interaction of *T. rubrum* conidia with peritoneal mouse macrophages. They found that the phagocytosis of *T. rubrum*, by the mouse macrophages, is inhibited by *T. rubrum* exoantigens. In addition, *T. rubrum* was able to down modulate MHC class 2 and stimulate the expression of IL-10. They noted that the fungi was able to differentiate into hyphae and kill the macrophages. This demonstrated that *T. rubrum* has mechanisms that are able to reduce the response of the mouse immune system [6].

A follow up study by Santiago *et al.* [6] aimed to reproduce these results in human subjects, while observing the interaction between the human dendritic cells, macrophages, and *T. rubrum*. Included in the study were ten healthy patients, between 40 and 70 years old, with a diagnosed *T. rubrum* infection, as well as ten non-infected individuals. Human macrophages and dendritic cells were infected with conidia. They were isolated and observed after different intervals of staining and were analyzed utilizing light microscopy. Analysis showed that the fungi differentiated into hyphae after 8 h of incubation with macrophages. However, in the presence of isolated dendritic cells, the conidia were not able to differentiate into hyphae. The viability of macrophages exposed to the hyphae decreased with time of culture, with the majority of macrophages dead after 10 h. Dendritic cells were not affected by the hyphae and remained viable after 10 h. A decrease in the phagocytic rate of conidia was observed after 30 min of exposure. The presence of exoantigens inhibited the phagocytic index in a dose dependent manner. ELISA analysis revealed that elevated levels of IL-12, IL-10, and TNF-α, were produced from the stimulated dendritic cells relative to the controls. Dendritic cells incubated with conidia were able to stimulate T cell proliferation, but those in healthy individuals were not [6].

De Oliviera, Vasconcellos, Sakai-Valente, Sotto, Luiz, Belda Júnior, De Sousa, Benard, and Criado [7] conducted a study on the role of toll like receptors 2 and 4 in localized dermatophytosis (LD) and disseminated dermatophytosis (DD). TLR2 and TLR4 are known to identify bacterial and fungal components and modulate the production of cytokines necessary in mounting an immune response. Seven patients with DD and eight with LD were selected. Twenty control skin samples taken from healthy individuals undergoing plastic surgery were used for comparison. Subjects were excluded if they had any comorbidity that could alter their immune function. *T. rubrum* was identified as the causative agent of the subjects’ infections, and the subjects had refrained from using any topical or systemic treatments for one month leading up to the study. A biopsy was taken using a standard punch biopsy technique. TLR2 and TLR4 were quantified using immunohistochemistry. Results revealed no significant difference between receptor expression in the LD and DD groups. There was a significant reduction in TLR4 expression in the upper and lower epidermis compared to the control group. No significant difference was found in TLR2 expression compared to the control group [7].

## 3. Results and Discussion

Garcia-Madrid *et al.*’s [1] study suggests that some human immune systems are unable to elicit a specific immune response necessary to combat *T. rubrum*. The authors showed that once the keratinocytes were exposed to whole conidia, essentially the immune system’s first line of defense was inactivated. This was due to *T. rubrum* manipulating the host defense, which allowed the conidia to down modulate receptors such as, TLRs, HBD-1, and HBD-2, which serve as necessary components of the initial host defense. The alterations to the innate immune system that were observed in their study are most likely, partially responsible for the chronic cases of dermatophytosis that are commonly seen worldwide [1]. Similarly, De Oliviera *et al.* [7] observed reduced expression in TLR4 in the epidermis of patients with dermatophotysis. TLR4 is important in the human immune response as it recognizes bacterial lipopolysaccharide and fungal O-linked mannans. Therefore, De Oliviera *et al.* suggested that down modulation of TLR4 may play a role in the mechanism that results in a decreased immune response to organisms like *T. rubrum* [7]. The results of both Garcia-Madrid *et al.*’s [1] and De Oliveria *et al.*’s [7] studies demonstrated that dermatophytoses, such as *T. rubrum*, are able to down modulate receptors (such as TLRs and HBDs) that are crucial in activating the appropriate immune response to various pathogens [1,7].

Waldman *et al.* [3] concluded that the immune system’s response to dermatophytes is mediated by cell-mediated immunity. In their study, patients with a chronic dermatophytosis infection exhibited depressed cytotoxic responses of T lymphocytes towards the dermatophytic antigen. However, in healthy patients the cytotoxic response of T lymphocytes proved to be effective and, in fact, did massive damage to the hyphae. Therefore, they suggested that more effective treatment modalities of tinea pedis may be related to the restoration of cell-mediated immunity [3]. Santiago *et al.* [6] also suggest the correlation between a cell-mediated immune response and an effective host defense against *T. rubrum*. The authors concluded that dendritic cells’ suppression of fungal growth and ability to stimulate T cells suggests that they play an important role in successfully coordinating the body’s cell-mediated immunity response [6]. While dendritic cells remained viable after exposure to the antigen, macrophages did not, thus compromising the phagocytic activity of the macrophages. Both Waldman *et al.* [3] and Santiago *et al.* [6] emphasized the importance of cell-mediated immune response to dermatophytoses [3,6].

Esquenazi *et al.*’s [5] study proved that *T. rubrum* expresses carbohydrate specific adhesions that recognize mannose and galactose, sugars that are present on the surface of host epithelial cells. This may be an integral component of *T. rubrum’s* ability to invade mammalian epithelial cells [5]. This is because recognizing a pathogen is the very first step in the immune response; such recognition is dependent upon the interaction between the host and the carbohydrate-binding proteins on the surface of an antigen. Bressani *et al.*’s [2] study indicated the possibility that, similar to certain strains of *Candida* and *Cryptococcus*, *T. rubrum* is able to evade the mannose receptors of dendritic cells and macrophages [2]. This provides a very important window into how the cellular immune response becomes defective. In a normal immune response, the innate immune system would use PRRs to recognize the PAMPs (which contain the mannans in the yeast), this would allow the macrophages and dendritic cells which contain a mannose receptor (MR) to release cytokines after interacting with the yeast. Ideally, this process would ultimately induce phagocytosis of the pathogen. However, Bressani’s study showed that some fungi contain the ability to evade the MR which would prevent the cytotoxic immune response and induce an infection. Bressani *et al.* [2] and Esquenazi *et al.* [5] discovered the importance of mannose and carbohydrate receptors in recognizing pathogenic dermatophytoses [2,5]. This manipulation to the cellular immune response seen in a chronic case of the infection ultimately inhibits the activation of T lymphocytes, a necessary component to the immune host defense.

## 4. Conclusions

Understanding the variety of complex immune responses to *Trichophyton* *rubrum* is essential to the treating physician in developing therapeutic strategies for those individuals who suffer from a chronic manifestation of tinea infections [4]. About a fifth of all *T. rubrum* skin infections will develop into a chronic state. *T. rubrum* colonizes the superficial layers of skin and causes common, but persistent infections such as “athlete’s foot”, onychomycosis in the nails, “jock itch” in the groin, and ringworm on any epidermal surface. Often, acute manifestations of *T. rubrum* may be treated successfully with a topical antifungal. However, in the chronic form, *T. rubrum* can invade the deeper levels of the epidermis, suppressing an immune response, and prolonging symptoms such as mild to intense itch, scaly pattern on the skin, and plaques and lesions. This type of dermatophyte can have severe implications for those suffering from a chronic infection, which often does not respond to treatment.

Exploring the mechanisms by which *T. rubrum* evades the initial host defense and manipulates the cellular immune response is vital to developing effective treatment modalities. A more successful treatment for chronic dermatophytoses would involve targeting the mechanisms of *T. rubrum* that deactivate the immune response, such as the ways in which it inhibits TLRs, and determining a way to restore the cell-mediated immune response, such as reactivating the phagocytic activity of the macrophages. More research needs to be conducted to determine the possible ways to reinstate these mechanisms, so that defective chain in the immune response may be targeted. A therapeutic intervention which achieves these goals, would significantly improve the quality of life for many people who suffer from chronic *T. rubrum* infections.
